# Determinants of COVID-19 vaccine uptake among Nigerians: evidence from a cross-sectional national survey

**DOI:** 10.1186/s13690-023-01107-1

**Published:** 2023-05-26

**Authors:** Temitope Olumuyiwa Ojo, Ayodeji Oluwole Ojo, Olajumoke Elizabeth Ojo, Bosede Olufunmilayo Akinwalere, Adebowale Femi Akinwumi

**Affiliations:** 1 Department of Community Health, Obafemi Awolowo University, Ile-Ife, Osun State +234 8035798224 Nigeria; 2 Department of Agricultural Economics, University of Ibadan, Ibadan, Nigeria; 3 Department of Agricultural Extension and Communications, Federal University of Technology, Akure, Nigeria; 4 Department of Community Medicine, Ekiti State University, Ado Ekiti, Nigeria

**Keywords:** COVID-19, Vaccine uptake, Determinants, Nigeria

## Abstract

**Background:**

COVID-19 ranks as one of the largest public health threats in recent times. It is associated with huge health, economic and social consequences. Although vaccination is an effective control measure, COVID-19 vaccine uptake has been suboptimal in many low/middle income countries. Hence this study assessed the factors influencing COVID-19 vaccine uptake among Nigerian households.

**Data and methods:**

This study analyzed secondary data from the COVID-19 High-Frequency Phone Survey of Households that was collected by the National Bureau of Statistics between November 2021 and January 2022. Relevant data were analyzed using descriptive statistical tools and the Multivariate Regression model.

**Results:**

Out of 2370 respondents, only 32.8% of the respondents were vaccinated against COVID-19. Respondents living in urban areas (34.4%) had a higher level of COVID-19 vaccine uptake relative to those living in rural Nigeria (30.9%). Results from the Multivariate Regression model revealed that adults aged ≥ 60 years (OR 2.20; *p* = 0.012), respondents with primary (OR 1.72; *p* = 0.032), secondary (OR 1.77; *p* = 0.025) and tertiary education (OR 3.03; *p* < 0.001), respondents with access to health insurance (OR 1.68; *p* = 0.004), those who obtained vaccine information from health workers (OR 3.92; *p* < 0.001), the government (OR 3.22; *p* < 0.001), and the mass media (OR 1.75; *p* = 0.003) were more likely to be vaccinated. Also, respondents living in North Central (OR 2.02; *p* < 0.001), North East (OR 1.48; *p* = 0.039), South West (OR 2.63; *p* < 0.001), and South South (OR 1.49; *p* = 0.031) regions had higher odds of being vaccinated.

**Conclusions:**

The study recommends increased media campaigns and advocacy for COVID-19 vaccination in the South East and North West regions. Persons with no formal education and younger persons aged 18–29 years should be targeted with COVID-19 vaccine-related information given that they were less likely to be vaccinated. Dissemination of relevant information through government sources, mass media and health workers is encouraged so as to positively influence decisions to receive COVID-19 vaccines among citizens.

## Introduction

The coronavirus disease 2019 (COVID-19) is a public health issue with adverse impacts on food security, livelihoods, and security globally [1] and the World Health Organization (WHO) officially declared the pandemic a public health emergency of international concern on the 30th of January 2020 [2]. The declaration ushered humanity into a new normal in which countries had to implement different control measures such as travel restrictions, phased lockdowns, a ban on public gatherings, and enforcement of mask use among others. These had huge socio-economic consequences. For instance, the COVID-19 lockdown periods were associated with a 34.1% economic loss amounting to USD 16 billion in Nigeria’s Gross Domestic Product (GDP) with services and agriculture sectors being the worst hit [3]. Furthermore, about 60% of Nigerians were food insecure and this may have worsened due to the adverse impact of COVID-19 [4]. Therefore, COVID-19 has multidimensional impacts on health, food security, and the economy [5].

Recent data from the World Health Organization (WHO) revealed that over 651 million cases and 6.6 million deaths are attributed to COVID-19 globally [6]. In Nigeria, 266,057 cases with 3,155 deaths have been reported [7]. However, the trend is being reversed with the introduction of COVID-19 vaccines across the globe. Researchers and several pharmaceutical firms have developed and introduced COVID-19 vaccines to minimize the risk of COVID-19 related deaths [8, 9]. Currently, the WHO has approved 11 vaccines for emergency use listing [10], while Nigeria has only approved seven vaccines for emergency use listing namely Vaxveria (Oxford/AstraZeneca), Covishield (Serum Institute of India), Comirnaty (Pfizer/BioNTech), Jcovden (Johnson and Johnson) and Spikevax (Moderna), Sputnik V (Gamaleya) and Covilo (Sinopharm) [11, 12].

Vaccination has proven to be an effective public health intervention in reducing the burden of infectious diseases across the globe [13, 14]. The vaccines are safe and effective in preventing serious or fatal cases of COVID-19 [15]. The COVID-19 vaccines have saved over 19 million lives across 185 countries thus reducing global deaths from COVID-19 by 63% [16]. However, the effectiveness of the COVID-19 vaccine in preventing and mitigating the adverse impact of the disease is constrained by widespread hesitancy [17, 18]. Vaccine hesitancy is defined as a behavior, resulting from several factors including lack of confidence (in vaccine or provider), complacency (does not perceive a need for a vaccine), and convenience (access issues) [21]. About 111 million doses of the COVID-19 vaccines have been administered in Nigeria as of February 5, 2023 [19]. Only 30.5% of the Nigerian population have full COVID-19 vaccination as of February 2023 [20]. This rate is low relative to Nigeria’s 211 million population, hence the country missed the WHO’s 70% vaccination coverage target by the end of June 2022 [8, 21, 22]. Although COVID-19 vaccines are available in-country, the underlying factors of why low vaccine uptake exists require further investigation.

The Andersen health behavior model was proposed by Andersen [26] and Andersen and Newman [27] to explain how and why people utilize specific type of health services or general types of health services. This model may be useful in understanding the factors that may influence COVID-19 vaccine uptake. Andersen assessed measures which include equity, quality of delivery and the environment (external or healthcare system) and how they impact access and utilization of healthcare services. The model has three main components namely: **“**predisposing factors” which refers to sociodemographic characteristics, while “enabling factors” comprise financial and organizational considerations that enable services utilization and lastly “need factors” entails perceived and evaluated need for health services. The Andersen health behavior model is quite relevant in explaining vaccines uptake, hence the reason it underpins this study.

Therefore, based on the Anderson’s model; this study assessed the “predisposing factors” influencing the uptake of the COVID-19 vaccine among a nationally representative sample of Nigerians. It is expected that the findings from this study will inform interventions aimed at improving COVID-19 vaccine uptake in Nigeria and fill important gaps in public health literature on this area of research.

## Materials and methods

### Sampling and data Collection

This study analyzed secondary data from the COVID-19 High-Frequency Phone Survey of Households that were collected by the National Bureau of Statistics between November 2021 and January 2022. Specifically, the data from Phase 2 Round 1 Sections A,2,5,6, 9a, and 12 of the phone surveys were analyzed in this study. The households were drawn from the sample of 4,976 households interviewed in 2018/2019 for Wave 4 of the General Household Survey Panel (GHS-Panel) which served as the sampling frame. The sample of households is nationally representative as data was collected across the six geopolitical zones. In every visit for the GHS-Panel, phone numbers are collected from up to 4 members of each household or two non-members through which respondents can be reached in future surveys.

A total of 3,000 households were selected from the frame of 4,934 using the balanced sampling approach to answer questions in this round of data collection. However, due to incomplete information for some variables, only 2,122 respondents (household heads) were included for this analysis.

The data provides information on the COVID-19 induced changes in food security, employment, income, access to basic services, coping strategies, and vaccination [23]. Approval to download and analyze the dataset was obtained from the World Bank.

### Data analysis

The data were analyzed using Stata version 15 (StataCorp, College Station, TX). Categorical variables were summarized using frequencies and percentages. Sampling weights were applied to all analyses and the unit of analysis was individuals (heads of household). The chi-square test was conducted to assess the association between categorical variables such as vaccine uptake versus sociodemographic characteristics. The outcome variable of interest was “ever received COVID-19 vaccine” which means the respondent has received at least one dose of the COVID-19 vaccine. Independent variables included age, place of residence (rural/urban), information sources about the COVID-19 vaccine, geopolitical zones, occupation of household head, access to health insurance, and Job loss due to COVID-19.

### The multivariable binary logistic regression model

The multivariable binary logistic regression model was used to assess predictors of COVID-19 vaccine uptake. The odds ratio and 95% confidence intervals were derived and level of statistical significance was set at 0.05.

Y_i_ = β_0_ + β_1_ × _1_ + β_2_ × _2_ + β_3_ × _3_ + β_4_ × _4_ +…. β_14_ × _14 +_ µ_i_.

### Model specifications

Y_i_= COVID-19 Vaccine uptake (Ever received vaccines, 1 = yes, 0 otherwise).

X_1_ = Age (years)

X_2_= Household size (Headcount)

X_3_ = Sex of household head (1 = Male, 0 otherwise)

X_4_ = Job loss arising arising from COVID-19 (1 = yes, 0 otherwise)

X_5_ = Rural location (1 = yes, 0 otherwise)

X_6_ = Source of health information (Health workers, family and friends, media)

X_7_ = Geopolitical zone (North East, North West, North Central, South East, South West, South South)

X_8_ = Occupation (Unemployed, Agriculture, Trading and Transport, Public Servant, and Others)

X_9_ = Educational status (None, Primary, Secondary, Tertiary)

## Results

A total of 2122 responses were analyzed and 1593 (66.6%) persons were unvaccinated. Most of the respondents (71.6%). were within the 30–59 years age bracket. Respondents who were less than 30 years old had the lowest vaccine uptake while those who were 60 years and above had the highest vaccine uptake. A higher proportion of females (68.4%) did not receive the COVID-19 vaccine.

Respondents who indicated that they received information about the COVID-19 vaccine from health workers, government sources as well as family and friends had higher vaccination uptake and this was statistically significant (p < 0.001). However, a lesser proportion of those who received information about the COVID-19 vaccine from family and friends were vaccinated. However, a statistically significant relationship existed between family and friends as a source of information and being vaccinated (p < 0.001). Across the geopolitical zones, the Southwest recorded the highest COVID-19 vaccine uptake (44.5%) whereas South East had the lowest uptake (21.4%). The study found a significant relationship between the geopolitical zones of residence and being vaccinated (p < 0.001). The majority (50.5%) of the respondents who had access to health insurance were vaccinated. A statistically significant relationship existed between having access to health insurance and being vaccinated (p < 0.001). Respondents who were public servants had the highest vaccination rates (38.4%). There was a statistically significant relationship between education and being vaccinated (p < 0.001) as more respondents (46.5%)x with tertiary education were vaccinated. (Table 1)

**Table 1:** Distribution of the respondents by vaccination status, 2022

Variables	Vaccinated (n = 708)	Non-vaccinated (n = 1,414)	Total (n = 2,122)	Statistical comparison
**Age (years)**				
< 30	17 (20.5)	66 (79.5)	83 (3.9)	χ^2^ = 21.554
30 — 59	506 (23.9)	1013 (47.7)	1519 (71.6)	**P < 0.001**
≥ 60	185 (35.6)	335 (64.4)	520 (24.5)	
**Sex**				
Male	633 (33.6)	1252 (66.4)	1,885 (88.8)	χ^2^ = 0.9743
Female	75 (31.6)	162 (68.4)	237 (11.2)	p = 0.509
**Household size**				
1 to 6	382 (32.7)	786 (67.3)	1,168 (55.0)	χ^2^ = 0.508
> 6	362 (33.2)	727 (66.8)	1,089 (51.3)	p = 0.476
**Place of residence**				
Urban	321 (35.3)	589 (64.7)	910 (42.9)	χ^2^ = 2.614
Rural	387 (31.9)	825 (68.1)	1,212 (57.1)	p = 0.106
**Information sources**:				
**Health workers**				
Yes	254 (48.6)	269 (51.4)	523 (24.7)	χ^2^ = 72.138
No	454 (28.4)	1,145 (71.6)	1,599 (75.4)	**p < 0.001**
**Government sources**				
Yes	226 (46.0)	265 (54.0)	491 (23.1)	χ^2^ = 46.079
No	482 (29.6)	1,149 (70.5)	1,631 (76.9)	**p < 0.001**
**Family and Friends**				
Yes	43 (19.2)	181 (80.8)	224 (10.6)	χ^2^ = 22.612
No	665 (35.0)	1,233 (65.0)	1,898 (89.4)	**p < 0.001**
**Media**				
Yes	60 (27.5)	158 (72.5)	218 (10.3)	χ^2^ = 3.729
No	648 (34.0)	1,256 (66.0)	1,904 (89.7)	p = 0.053
**Geopolitical zones**				
North Central	135 (39.9)	203 (60.1)	338 (15.9)	
North East	116 (34.3)	222 (65.7)	338 (15.9)	χ^2^ = 62.777
North West	77 (25.8)	221 (74.2)	298 (14.0)	**p < 0.001**
South East	78 (21.4)	286 (78.6)	364 (17.2)	
South South	108 (31.0)	240 (67.0)	348 (16.4)	
South West	194 (44.5)	242 (55.5)	436 (20.6)	
**Respondent had Job loss arising from COVID-19**				
No	499 (34.6)	945 (65.4)	1,444 (68.1)	χ^2^ = 2.889
Yes	209 (30.8)	469 (69.2)	678 (32.0)	p = 0.089
**Access to health insurance**				
No	615 (31.7)	1323 (68.3)	1,938 (91.3)	χ^2^ = 26.742
Yes	93 (50.5)	91 (48.7)	184 (8.7)	**p < 0.001**
**Occupation**				
Unemployed	109 (31.9)	233 (68.1)	342 (16.2)	
Agriculture	238 (33.1)	481 (66.9)	719 (33.9)	
Trading and Transport	142 (30.5)	324 (69.5)	466 (22.0)	χ^2^ = 7.082
Public Servant	168 (38.4)	270 (61.6)	438 (20.6)	p = 0.132
Others	51 (32.5)	106 (67.5)	157 (7.4)	
**Education**				
No education	28 (18.7)	122 (81.3)	150 (7.1)	χ^2^ = 65.449
Primary education	182 (29.4)	436 (70.6)	618 (29.1)	**p < 0.001**
Secondary education	241 (30.1)	560 (69.9)	801 (37.8)	
Tertiary education	257 (46.5)	296 (53.5)	553 (26.1)	

### Determinants of COVID-19 vaccine uptake among nigerian households

The factors influencing COVID-19 vaccine uptake in Nigerian households are presented in Table 2. The results revealed that heads of households aged ≥ 60 years (OR 2.20; 95% CI 1.19, 4.07; *p* = 0.012) had higher odds of being vaccinated compared to those less than 30 years. Respondents with access to health insurance were more likely to be vaccinated compared to those without access to health insurance (OR 1.68; 95% CI 1.18, 2.318; *p* = 0.004). Head of households that received COVID information from health workers had higher odds (OR 3.92; 95% CI 2.99, 5.13; *p* < 0.001) of getting vaccinated. Similarly, heads of households that received COVID information from government sources had higher odds (OR 3.22; 95% CI 2.44, 4.23; *p* < 0.001) of being vaccinated relative to those who did not. Households whose source of COVID information was from mass media, were more likely to get vaccinated (OR 1.75; 95% CI 1.21, 2.53; *p* = 0.003). Households living in the North Central (OR 2.02; 95% CI 1.37, 2.99; *p* < 0.001), North East (OR 1.48; 95% CI 1.02, 2.15; *p* = 0.039), South south (OR 1.49; 95% CI 1.04, 2.13; *p* = 0.031) and South West zones (OR 2.63; 95% CI 1.76, 3.94; *p* < 0.001) had higher odds of being vaccinated relative to their counterparts in the North West. Also, respondents with primary education (OR 1.72; 95% CI 1.05, 2.83; *p* = 0.032), secondary education (OR 1.77; 95% CI 1.08, 2.92; *p* = 0.025) and tertiary education (OR 3.03; 95% CI 1.83, 5.01; *p* < 0.001) were more likely to be vaccinated compared to their counterparts with no formal education.

**Table 2:** Determinants of COVID-19 Vaccine Uptake among Nigerian Households, 2022

Variables	Odds Ratio	95% CI	P- values
**Age of household head**			
< 30 years	Ref		
30–59 years	1.77	0.99, 3.17	0.056
≥ 60 years	**2.20**	**1.19, 4.07**	**0.012**
**Household size**			
1–6 people	Ref		
> 6 people	1.21	0.97, 1.51	0.087
**Sex of household head**			
Female	Ref		
Male	0.86	0.62, 1.19	0.355
**Job loss arising from COVID-19**			
No	Ref		
Yes	0.93	0.75, 1.16	0.540
**Rural location**			
No	Ref		
Yes	1.23	0.98, 1.56	0.079
**Access to health insurance**			
No	Ref		
Yes	**1.68**	**1.18, 2.38**	**0.004**
**Source of Information about COVID-19**:			
**Health workers**			
No	Ref		
Yes	**3.92**	**2.99, 5.13**	**< 0.001**
**Government sources**			
No	Ref		
Yes	**3.22**	**2.44, 4.23**	**< 0.001**
**Family & friends**			
No	Ref		
Yes	1.06	0.71, 1.57	0.790
**Mass Media**			
No	Ref		
Yes	**1.75**	**1. 21, 2.53**	**0.003**
**Geopolitical Zone**			
North West	Ref		
North Central	**2.02**	**1.37, 2.99**	**< 0.001**
North East	**1.48**	**1.02, 2.15**	**0.039**
South West	**2.63**	**1.76, 3.94**	**< 0.001**
South East	1.02	0.67, 1.55	0.938
South South	**1.49**	**1.04, 2.13**	**0.031**
**Occupation**			
Unemployed	Ref		
Agriculture	1.17	0.86, 1.59	0.330
Trading and Transport	1.07	0.77, 1.49	0.692
Public Servant	1.15	0.82, 1.61	0.414
Others	1.14	0.72, 1.74	0.632
**Education**			
No Education	Ref		
Primary Education	**1.72**	**1.05, 2.83**	**0.032**
Secondary Education	**1.77**	**1.08, 2.92**	**0.025**
Tertiary Education	**3.03**	**1.83, 5.01**	**< 0.001**

## Discussion

The need to investigate the factors influencing COVID-19 vaccine uptake in Nigeria cannot be overemphasized. An understanding of the determinants of uptake will guide policymakers in prioritizing effective communication channels, addressing the concerns of the citizens, and ensuring easy access to vaccines. This study reported that about two-thirds of the respondents (67%) did not obtain the COVID-19 vaccine. This is consistent with the earlier findings of Soares et al. in Portugal which reported that 56% of the respondents wanted to delay and 9% outrightly refused COVID-19 vaccination [24]. The low levels of COVID-19 vaccine uptake may hamper pandemic control efforts in Nigeria more so the country missed the 70% vaccine coverage target by June 2022 recommended by the WHO [22].

Generally, the uptake of the COVID-19 vaccine can be linked to the awareness level, information sources, and perception of individuals [25]. The main information sources were family and friends, government, media, and health workers. This is consistent with the earlier findings of Faye and colleagues across some West African counties where they reported that important information sources were family and friends and media [26].

Our study reported that respondents who received COVID-19 vaccine information from health workers, government sources and media had significantly higher odds of being vaccinated. This may suggest that these three information sources were probably the most trusted among the available information sources by the citizenry. In addition, health workers and government sources were more likely to provide reliable information on vaccines to the general public, especially in this era of pandemic-related misinformation. Having more exposure to information on the COVID-19 vaccines from credible sources makes individuals more likely to get vaccinated [27].

The study found a positive and statistically significant relationship between age and COVID-19 vaccine uptake. Persons aged 60 years or more were more likely to be vaccinated against COVID-19 and this could be linked to the fact that older persons were more likely to have severe symptoms if they contract the disease [28, 29]. Hence, they may appreciate the urgent need to get vaccinated. This is consistent with the findings of many other studies which reported that younger people were less keen to take the vaccine as compared to older persons [9, 24, 30, 31]. However, it should be noted that at the time of the survey, vaccination was recently extended to all adults (18 years or older) in the country [32].

Education was a significant factor associated with vaccine uptake in this study as educated respondents were more likely to be vaccinated. This is not unexpected as education has been reported as a significant predictor of COVID-19 vaccination in some past studies [33, 34]. In addition education is a major social determinant of health since it influences individuals’ perception of risk, health seeking behavior and utilization of health services [35].

The study also found a direct and statistically significant association between access to health insurance and the likelihood of being vaccinated. Specifically, household heads with access to health insurance were twice as likely to be vaccinated when compared to their counterparts with no access to health insurance. This can be linked to the fact that most people in Nigeria who have access to health insurance are educated, as either themselves or a family member works in the formal sector of the Nigerian economy. This implies that such households may have better access to credible information on the prevention and adverse impacts of COVID-19 that may encourage them to take the vaccine.

Across the geopolitical zones, it appears that respondents from the North Central, North East, South West, and South south regions had higher odds of being vaccinated. This geographical disparity in vaccine uptake has huge public health implications. To address this challenge, more mobilization and community engagements are required in the other regions as a part of measures to address the low level of vaccine uptake across the country.

Based on the findings of this study, the following recommendations are suggested. Firstly, Government and development partners should increase media campaigns and empower health workers to intensify advocacy for COVID-19 vaccination. Secondly, the government should increase access to health insurance given that the surveyed households with insurance coverage were less likely to be hesitant to get vaccinated. Lastly, the Nigerian youths who constitute a majority of the population should be targeted with COVID-vaccine related information aimed at improving their vaccine uptake. Lastly, for future research, it is important to conduct a qualitative study that will assess the social drivers of vaccine hesitancy among Nigerians.

### Limitations

The study was prone to social desirability bias as some respondents may have indicated that they had received vaccines while they did not. Also, selection bias is another limitation that may characterize phone-based surveys because some groups of persons without phones are naturally excluded from interviews.

## Data Availability

The dataset used for this study is available at https://microdata.worldbank.org/index.php/catalog/3712.

## Abbreviations

CI: Confidence Interval
COVID-19: Corona Virus Disease 2019
GDP: Gross Domestic Product
GHS-Panel: General Household Survey-Panel
SARS-CoV2: Severe Acute Respiratory Syndrome- Coronavirus 2
UN: United Nations
WHO: World Health Organization
